# Efficacy of and Satisfaction with an In-house Developed Natural Rubber Cardiopulmonary Resuscitation Manikin

**DOI:** 10.5811/westjem.2019.10.43004

**Published:** 2019-12-09

**Authors:** Sittichoke Anuntaseree, Ekwipoo Kalkornsurapranee, Varah Yuenyongviwat

**Affiliations:** * Prince of Songkla University, Department of Orthopedics, Faculty of Medicine, Songkhla, Thailand; † Prince of Songkla University, Department of Materials Science and Technology, Faculty of Science, Songkhla, Thailand

## Abstract

**Introduction:**

A barrier to cardiopulmonary resuscitation (CPR) training in low-income countries is limited resources. Our goal was to build a CPR training model of simple design that would provide a good feedback system.

**Methods:**

We developed a low-cost, Basic Life Support training manikin made entirely of natural rubber. Our in-house manikin provides feedback when performing correct chest compression and rescue breathing. The properties of the manikin were tested using simulated chest compression in a laboratory and compared with a commercial manikin. Forty healthy nurse volunteers with CPR experience performed CPR in both types of manikins and responded to questionnaires.

**Results:**

A tensile test in a laboratory demonstrated that both types of manikins had acceptable ranges of properties for real-situation CPR in cardiac arrest patients. There were no differences in aesthetic properties, and the manikins felt to the volunteers like a real patient when they were performing chest compression. The feedback response was clear when chest compressions and rescue breathing were performed correctly, and the overall satisfaction with the manikin was good. In addition, the mean scores in terms of the manikin feeling like a real patient when performing rescue breathing and the positive feedback from the rubber manikin were statistically higher than those for the commercial manikin (p=0.001 vs. p=0.023).

**Conclusion:**

The in-house developed CPR manikin employing real-time feedback by simple mechanics is effective compared with a commercial manikin. The advantage of our manikin is that it is easy to build and costs substantially less than a commercial manikin. The use of an in-house developed manikin could make effective CPR training more available in limited-resource areas.

## INTRODUCTION

Cardiopulmonary resuscitation (CPR) is a lifesaving skill used in many situations such as drowning and acute cardiac arrest due to heart disease. Basic CPR, which is performed with chest compressions and rescue breathing, increases the chance of survival.^1^ The CPR technique performed properly can save lives, especially within four minutes after the cardiac arrest.^2^,^3^ A study reported that the early initiation of CPR by bystanders was associated with a good outcome.^4^ Therefore, everyone should have the proper knowledge and skills to perform CPR, especially physicians, nurses, and paramedics.

The key to success of CPR is proper training, which provides the learner with Basic Life Support (BLS) knowledge and the opportunity to practice the needed skills. Skill development is achieved by practicing CPR on manikins that simulate the human body. Over the past 50 years, the number of CPR training sessions for the general public has increased.^5^ A study reported that 65% of the population in the United States (U.S.) had received CPR training at some point in their lifetime.^6^ However, one study in Pakistan reported that only 37% of medical students had even a basic knowledge of CPR.^7^ We wanted to produce a cost-effective CPR training model of simple design that could teach proper CPR with a good feedback system that responded to the correct CPR maneuvers. Our aim is to make CPR manikins more available in limited-resource areas.

## METHODS

### Design

We developed an in-house, low-cost, BLS training manikin made entirely of natural rubber. The manikin provides feedback when the user performs chest compressions and rescue breathing correctly. We are making this [patent-pending] intellectual property available at no charge for non-profit proposes to those who request it from us.

**Figure 1:**
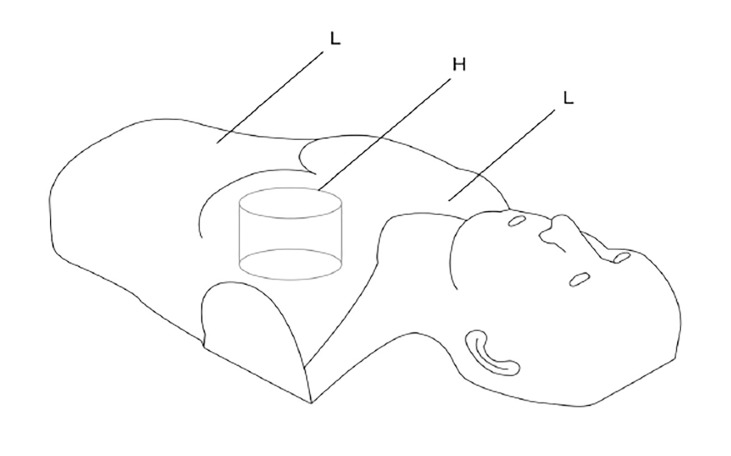
The cardiopulmonary resuscitation manikin consists of a high-density and highly elastic rubber to serve as the heart (H), and low-density foam rubber to form the head, neck, and body (L).

A durable, natural rubber film sheathes the outside of the manikin, which is the same size as an adult body from head to waist. The inside of the manikin is made from natural rubber foam that consists of two types of material: 1) the first material, in the center of the chest, is a very elastic, high-density rubber that simulates the heart;; and 2) the remainder of the manikin is made of low-density natural rubber foam to form the head, neck, and body (Figure 1).

**Figure 2:**
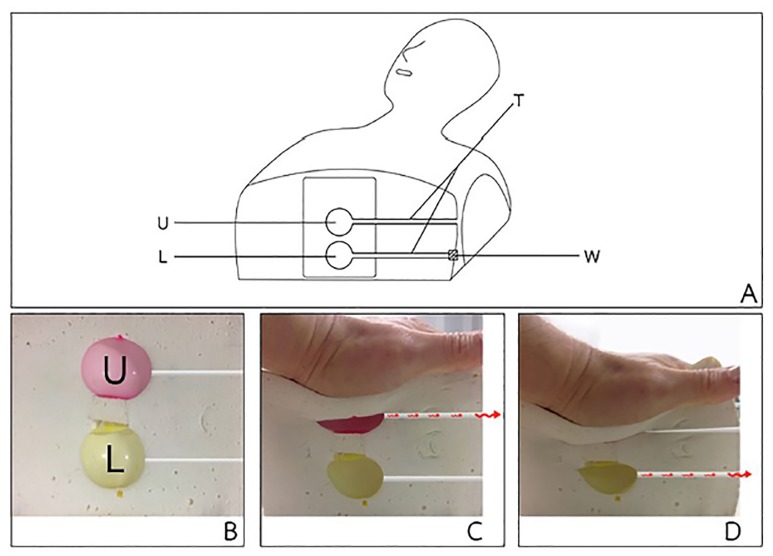
A, B, C, and D show the rubber foam cardiac mechanism: A) U = upper air space, L = lower air space, T = tube, W = whistle; B) cross-section of the pressure-sensing mechanism, U = upper air space, L = lower air space; C) beginning of chest compression; D) 1–2 inch depth of chest compression.

Inside the rubber that forms the heart, a twin air-space mechanism reacts to chest compressions. As chest compression is performed, the upper air space collapses. At a chest compression depth of 1–2 inches, the bottom air space collapses and releases air through a tube that leads to the outside of the manikin, and a whistle at the opening makes a sound (Figure 2).

**Figure 3:**
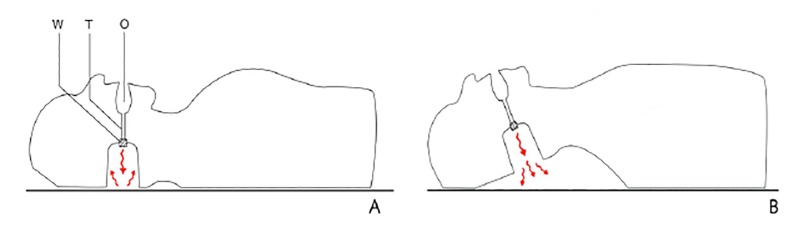
A and B show the mechanism that senses the breathing: O = oral cavity; T = tube; W = whistle. A) Blowing into the mouth without the chin lift maneuver limits the sound from the manikin; B) A loud whistle sound is produced when blowing into the mouth after the chin lift maneuver.

The mouth of the manikin is open. Its back and the back of the head are flat. The neck is curved and concave with a tilted occiput. In the innermost part of the manikin’s oral cavity, a tube connects the mouth to a space at the back of the manikin’s head where a whistle is installed. The whistle makes a sound while the user is blowing into the mouth of the manikin (Figure 3). The detailed process of building this manikin is described in the Appendix.

**Population Health Research Capsule**

What do we already know about this issue?

*Cardiopulmonary resuscitation (CPR) skill development is achieved by practicing CPR on manikins that simulate the human body*.

What was the research question?

*Our goal was to build a low-cost CPR training model of simple design that would provide a good feedback system*.

What was the major finding of the study?

*We designed an in-house developed manikin for CPR training that provides real-time feedback using simple mechanics and has a low manufacturing cost*.

How does this improve population health?

*The use of our in-house developed manikin could make effective CPR training more available in limited-resource areas*.

### Compression Test

**Figure 4:**
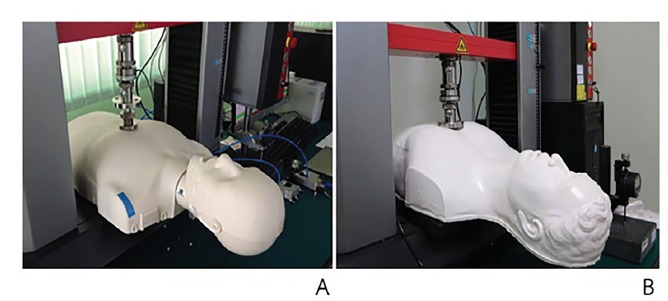
Prestan adult manikin (A) and natural rubber manikin (B).

**Figure 5:**
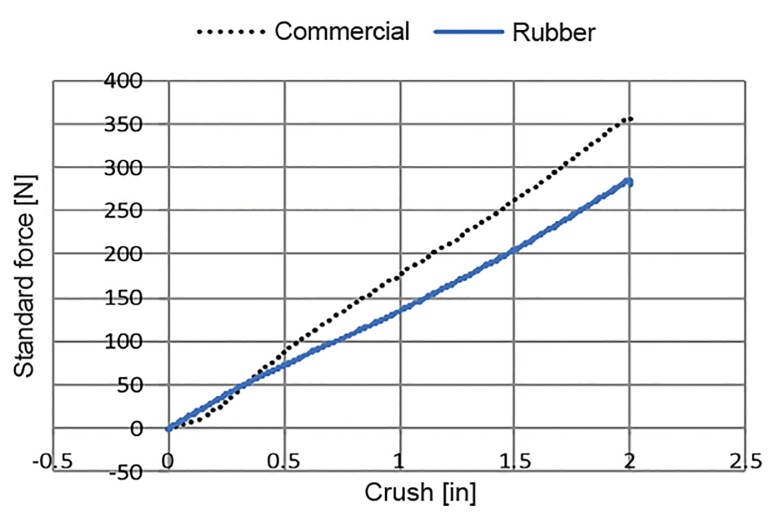
Tensile test results of simulated chest compression.

This study was approved by the Ethics Committee and the Institutional Review Board of the Faculty of Medicine, Prince of Songkla University, Thailand. The natural rubber properties of the CPR manikin were tested in a laboratory using a simulated chest compression, universal testing machine (Zwick/Roell Z010; Zwick GmbH & Co, Ulm, Germany). To evaluate the tensile strength of the materials, chest compression was performed on both a commercial manikin (Prestan Adult Manikins, Prestan Products, LLC, Ohio, USA) and the natural rubber manikin that we developed in-house. The testing demonstrated that when both manikins (Figure 4) were compressed to a depth of 1.5 inches, the materials of both were in an acceptable range of a real CPR situation in cardiac arrest patients (155–443 Newtons in males, and 123–327 Newtons in females)^8^ (Figure 5).

### CPR Test

Forty healthy nurses, aged 20–50 years, volunteered for the CPR test. All of them had real-life CPR experience and no morbidities that would have limited their performance of CPR on the manikins. The volunteers were randomized into two groups by opaque envelopes containing a computer-generated sequence. The first group of 20 volunteers performed CPR on the rubber manikin using both chest compression and rescue breathing at a ratio of 30:2 (5 cycles), and then did exactly the same on the commercial manikin. The second group of 20 volunteers performed CPR following the same steps as the first group but performed CPR on the commercial manikin first and then on the natural rubber manikin. We collected data from the volunteers using a self-reported questionnaire on which they responded to questions about the appearance, response, and feedback of the two manikins, as well as the volunteers’ overall satisfaction with the manikins. The questionnaire for each manikin was completed immediately after CPR on each manikin.

### Statistical analysis

We performed the statistical analysis using the R software version 3.1.0 (R Foundation for Statistical Computing, Vienna, Austria). Using Student’s *t*-test, we analyzed the continuous data of the rubber manikin and the imported commercial manikin to detect differences. Statistical significance was assumed if p<0.05.

## RESULTS

The feedback data of the volunteers are shown in Table 1. There were no differences in terms of appearance with users reporting that the manikin felt like a real patient while they were performing chest compression. The volunteers also reported satisfaction with the in-house manikin’s positive feedback response when chest compression was correctly performed, and they reported overall satisfaction with the manikin (p=0.42, 0.83, 0.88, and 0.12, respectively). However, the mean score regarding the manikin feeling like a real patient while performing rescue breathing was statistically significantly higher for the in-house developed manikin (p = 0.001), as was the mean score of positive feedback (p = 0.023).

## DISCUSSION

Effective CPR increases the chance of survival in cardiac arrest patients by two- to three-fold if CPR is done immediately after cardiac arrest.^9^–^12^ Participation in a CPR training program is key to successfully learn and/or improve the skills of healthcare workers. But it is also important that the general population have the knowledge and skills to perform CPR, in the event that a bystander is the first responder. To this end, many learning tools have been introduced over the last decade including self-instruction^13^ and simulation.^14^ To improve the quality of training, a manikin needs to feel like a real human and provide a real-time feedback system when the learner is performing CPR.^15^ Our study reports the results of the testing of the properties and performance of an in-house developed, rubber CPR manikin. The tensile strength was tested using a chest compression mechanism. Nurses with CPR experience performed chest compressions and rescue breathing on both the commercial, imported manikin and the rubber manikin.

Many manikins on the market have electronic systems to provide real-time feedback when users apply the correct chest compression force. The feedback systems include a computer monitor screen or a light that indicates correct compression. In rescue breathing, some manikins use chest expansion as a feedback response. In our study, we developed a low-cost CPR manikin of simple design. It provides real-time feedback responses via whistling sounds when the trainee performs chest compression and rescue breathing correctly. The sounds are caused simply by air passing through a whistle, a simple design that substantially reduces the cost of manufacturing. We believe that the development of this low-cost CPR manikin can expand or make CPR training more readily available in areas or countries with limited resources.

The cost breakdown of the rubber manikin can be categorized into the price of the raw materials, the fiberglass mold, and the fabrication of the rubber foam. The raw material is latex, which is not expensive and is readily available in many countries. Moreover, the fiberglass mold is simple and cheap. Lastly, the fabrication of rubber foam is not complicated; it does not require a lot of technical knowhow or technologically advanced equipment and/or facilities. Furthermore, the technology required for the production of this manikin can be easily transferred to small and medium-size enterprises. In our setting, the cost of the low-fidelity natural rubber CPR manikin under study was about 100 U.S. dollars. Meanwhile, the cost of the commercial product used for comparison in this study was about 400 U.S. dollars.

## LIMITATIONS

Some limitations of this study need to be acknowledged. First, we performed the comparison with only one type of commercial CPR manikin that was available in our hospital. The costs of other commercial products in Thailand are shown in the appendix. Second, this study could not blind the volunteers to the manikins tested (ie, they were aware of which manikin was which).

## CONCLUSION

We designed a low-cost manikin for CPR training that provides real-time feedback using simple mechanics and has a low manufacturing cost. We believe that, based on this model for creating a low-cost manikin, this concept could be expanded on for other training venues.

## Figures and Tables

**Table 1:** Results of simulated cardiopulmonary resuscitation (CPR) from both types of manikin.

	Rubber	Commercial	P value
Appearance			
Aesthetic properties	4.18 (0.71)	4.05 (0.78)	0.42
Chest compression			
Feels like a real patient	4.15 (0.66)	4.13 (0.76)	0.83
Response when performing correct CPR	3.98 (0.77)	4.00 (0.75)	0.88
Rescue breathing			
Feels like a real patient	4.13 (0.69)	3.65 (0.77)	0.001
Response when performing correct CPR	4.18 (0.81)	3.80 (0.85)	0.023
Overall satisfaction	4.10 (0.59)	3.90 (0.71)	0.12

Data are presented as mean and standard deviation.
